# *In vitro* patient-derived 3D mesothelioma tumor organoids facilitate patient-centric therapeutic screening

**DOI:** 10.1038/s41598-018-21200-8

**Published:** 2018-02-13

**Authors:** Andrea R. Mazzocchi, Shiny A. P. Rajan, Konstantinos I. Votanopoulos, Adam R. Hall, Aleksander Skardal

**Affiliations:** 1 Wake Forest Institute for Regenerative Medicine, Wake Forest School of Medicine, Medical Center, Winston-Salem, NC 27101 USA; 2 Virginia Tech-Wake Forest School of Biomedical Engineering and Sciences, Wake Forest School of Medicine, Medical Center Boulevard, Winston-Salem, NC 27157 USA; 3 Department of Surgery-Surgical Oncology, Wake Forest Baptist Medical Center, Medical Center Boulevard, Winston-Salem, NC 27157 USA; 4 Comprehensive Cancer Center at Wake Forest Baptist Medical, Medical Center Boulevard, Winston-Salem, NC 27157 USA; 5 Department of Cancer Biology, Wake Forest School of Medicine, Medical Center Boulevard, Winston-Salem, NC 27157 USA

## Abstract

Variability in patient response to anti-cancer drugs is currently addressed by relating genetic mutations to chemotherapy through precision medicine. However, practical benefits of precision medicine to therapy design are less clear. Even after identification of mutations, oncologists are often left with several drug options, and for some patients there is no definitive treatment solution. There is a need for model systems to help predict personalized responses to chemotherapeutics. We have microengineered 3D tumor organoids directly from fresh tumor biopsies to provide patient-specific models with which treatment optimization can be performed before initiation of therapy. We demonstrate the initial implementation of this platform using tumor biospecimens surgically removed from two mesothelioma patients. First, we show the ability to biofabricate and maintain viable 3D tumor constructs within a tumor-on-a-chip microfluidic device. Second, we demonstrate that results of on-chip chemotherapy screening mimic those observed in subjects themselves. Finally, we demonstrate mutation-specific drug testing by considering the results of precision medicine genetic screening and confirming the effectiveness of the non-standard compound 3-deazaneplanocin A for an identified mutation. This patient-derived tumor organoid strategy is adaptable to a wide variety of cancers and may provide a framework with which to improve efforts in precision medicine oncology.

## Introduction

**Figure 1:**
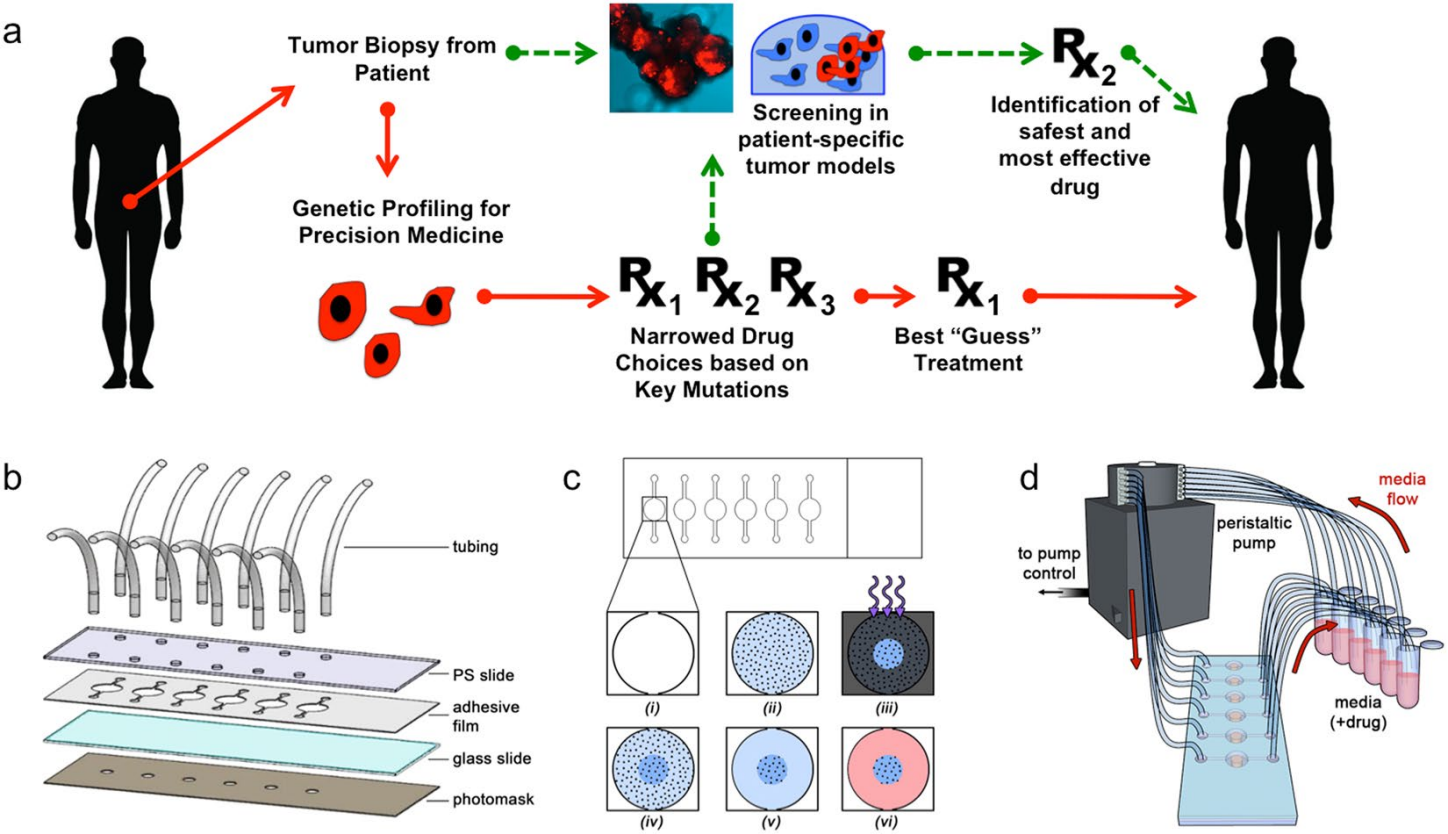
Patient-derived tumor-on-a-chip models for personalized precision medicine. (**a**) The concept of using engineered 3D *in vitro* models to better inform precision medicine treatment regimens on a patient-by-patient basis. Red arrows: The current state of the art precision medicine pipeline, in which treatments are identified for patients based on their tumor genetic profiles. In practice, even after identification of key mutations, oncologists are often left with several potential drug options, resulting in a best guess of the optimal treatment. Green arrows: Implementation of organoids created with patient cells can supplement genetic screens of biopsied tumor cells, ultimately predicting the optimal therapies for patients. (**b**) Schematic assembly of microfluidic device (see text). The patterned adhesive film both attaches surrounding layers together and forms the channels and chambers in which organoids are formed. (**c**) *In situ* organoid patterning technique: a microfluidic chamber (i) is filled with a mixture (blue) containing hydrogel precursors, photoinitiator, and patient-derived tumor cells (ii) and then illuminated with UV light through a photomask (gray) (iii). Exposed precursor is crosslinked into a hydrogel (dark blue), detaining cells within the region (iv), and non-crosslinked gel is flushed form the chamber with clean PBS from the chamber (v). Finally, PBS is replaced with DMEM (red) (vi) for incubation. (**d**) The total measurement set-up, featuring a low-volume, closed loop fluidic circuit for each organoid facilitated by a computer-controlled peristaltic pump.

Resistance to anti-cancer drugs is one of the most critical challenges in clinical oncology. The roots of resistance are most often ascribed to tumor heterogeneity, genetic mutation, oncogenic amplification, and changes in protein expression that influence the uptake, metabolism, and removal of drugs from the cell. As a result, resistance can arise dynamically during treatment and has been observed with every major anti-cancer agent. Chemotherapy decisions traditionally have been made based on statistical success rate of a drug for clinical patient populations, but not on how that drug affects the tumor of a specific patient. Precision, or personalized, medicine (PM) has emerged as a response to these challenges. Here, a tumor biopsy is profiled genetically to identify mutations that may indicate drugable targets^1–3^. Subsequently, if FDA-approved drugs or ongoing clinical trials are identified that address one of these targets, the therapy for that patient can be adjusted accordingly (Fig. 1, red arrows). However, the non-monolithic nature of tumors and the sheer complexity of the various factors that can be at play individually or in unison in the patient have made such data-driven prognostication both daunting and varying in effectiveness. In practice, the benefits of precision medicine are still ambiguous^4^. Even after identification of targeted mutations, oncologists are left with several drug options and with no way to predict if a particular treatment will be more beneficial to the patient than another. As such, even in cases where “actionable” mutations are identified, modification of a predetermined fixed treatment strategy is rare, given the unknown impact of that mutation on tumor biology and the equally unknown effect of chemotherapy options on a specific cellular phenotype.

As a potential improvement (Fig. 1a, green arrows), the same biospecimen could also be employed for screening of multiple candidate drug agents to determine effectiveness *in vitro*. Consequently, there is a tremendous need for accurate model systems to help predict response to anti-cancer drugs in individual patients^5,6^. However, tumor models have been limited by an inability to accurately replicate both progression and signaling mechanisms of cancer in a controlled environment. Animal models allow limited manipulation and study of these mechanisms, but are not necessarily predictive of results in humans^7^. Meanwhile, traditional *in vitro* 2D cell culture fails to recapitulate the 3D microenvironment of *in vivo* tissues^8^; in these planar systems, drug diffusion kinetics vary dramatically and cell-cell/cell-matrix interactions are inaccurate. Moreover, drug doses found to be effective in 2D cultures are frequently ineffective when applied to patients^9,10^. 3D cell culture has been shown to better reproduce *in vivo* effects, and as a result, are more accurate systems for *in vitro* cancer modeling. For example, we recently demonstrated that metastatic colorectal cancer (CRC) cells appear epithelial in conventional 2D tissue culture dishes, but take on the expected mesenchymal and metastatic phenotype when transitioned into a 3D liver organoid environment^11^. Additionally, integration of bioengineering^12,13^ with microfluidics has further resulted in organ-on-a-chip technologies for accurate and addressable testing of compact 3D organoids in parallel. These platforms can combine a variety of important parameters that permit better mimicry of *in vivo* conditions, including 3D architecture, cell-cell/cell-matrix interactions, circulation, and integration of multiple tissues within one platform, and have been used for drug testing^14^, toxicology^15^, high throughput screens, and disease modeling^16^. However, to date, because primary tumor cells have been difficult to maintain *in vitro*, such technologies have not been widely applied to patient cells, where the resulting analyses could be used to supplement genetic screens or ultimately allow robust prediction of optimal therapies.

Here, we present a report of drug screening with 3D organoids incorporating patient-derived tumor cells. For this, we utilize a 3D extracellular matrix-inspired hydrogel system^17–22^ that supports a biofabrication procedure that can be integrated with a microfluidic delivery system. This general approach has been applied previously to yield a broad collection of organoids that model the structure and function of *in vivo* tissues and serve as hosts for tumor constructs^11,15,18,23–26^. Using cells derived from mesothelioma tumor biospecimens from two patients, we demonstrate formation of 3D mesothelioma tumor organoids of high cellular viability and then perform a series of drug exposures. First, we demonstrate that this system can recapitulate patient drug response to chemotherapy, and then we probe the effects of an experimental drug compound, driven by patient-specific genetic analysis that identified a targetable mutation. These results demonstrate the efficacy of integrated personalized tumor organoids for chemotherapy drug screening and suggest the potential of the system to help optimize treatments for cancer patients.

## Results

### Biospecimen processing, organoid production, and assessment of patient-specific tumor organoids

Tumor tissues were obtained from two subjects during the course of standard treatment. Subject 1 was a 50-year-old male who was diagnosed with epithelioid malignant peritoneal mesothelioma. Precision medicine analysis of the tumor revealed two genomic alterations (BAP1 splice site 1729 + 1 G > A and PBRM1 N258fs*6) for which no targeted drugs or clinical trials were available. Due to the excessive volume of disease, the subject was referred for neoadjuvant systemic chemotherapy. Treatment was conducted with 6 cycles of cisplatin-gemcitabine followed by a single cycle of carboplatin-gemcitabine due to concerns of cisplatin-associated ototoxicity. Excellent clinical response to cisplatin-based chemotherapy was observed, with almost complete resolution of malignant ascites on a repeat 3 month re-staging CT. Subsequently, a cytoreductive surgery (CRS) with cisplatin-based heated intraperitoneal chemotherapy (HIPEC) was performed. During CRS, a segment of the tumor was resected, placed on ice, and transferred fresh to the lab for tumor organoid development. Importantly, the subject remains without radiologic evidence of disease 6 months after the operation without adjuvant treatment.

Subject 2 was a 38-year-old female who was also diagnosed epithelioid peritoneal mesothelioma. She was treated with 4 cycles of cisplatin and pemetrexed with mixed response followed by 2 CRS without HIPEC. During CRS, a segment of the omental tumor was resected, and treated as described for the previous tumor biospecimen. Final pathology indicated that, rather than epithelioid peritoneal mesothelioma, the tumor was actually well-differentiated papillary-cystic mesothelioma. Precision medicine analysis of the tumor revealed no targetable genomic alterations. The patient remains without radiologic evidence of disease 6 months after her operation without adjuvant treatment.

The tumor biospecimens were kept on ice following retrieval and delivered to the lab for cell processing within one hour. Following dissociation of the extracellular matrix, isolated tumor cells were mixed with a photopolymerizable hyaluronic acid (HA) and gelatin hydrogel precursor and introduced inside an adhesive film-based microfluidic device with multiple, independent sets of channels (Fig. 1b). A tumor construct was biofabricated in each circular chamber of the device by patterning through an integrated photomask (Fig. 1c), similar to our previous work^15^. Following flushing of unexposed precursor/cell mixture from the device, discrete 3D patient-derived mesothelioma constructs remained in each channel, after which media was continuously flowed from independent reservoirs by tubing connected to a micro-peristaltic pump (Fig. 1d).

**Figure 2:**
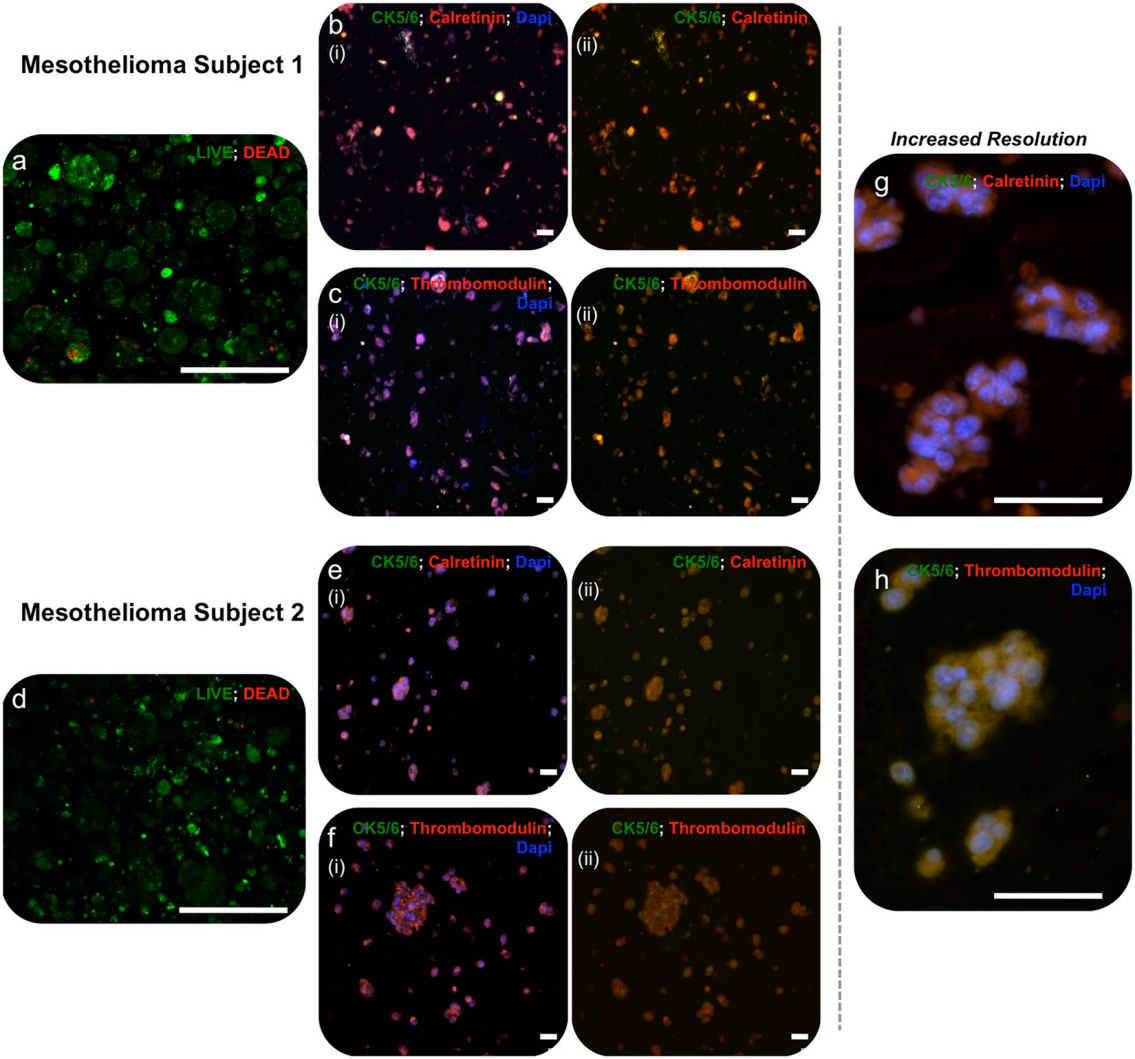
Patient tumor construct histological assessment. Histological assessment of patient-derived mesothelioma organoids from both subjects after seven days of incubation in DMEM. (**a**,**d**) LIVE/DEAD-stained organoids. Green – calcein AM-stained viable cells; Red – ethidium homodimer-stained dead cells. (**b**,**e**) Cytokeratin 5/6 and calretinin double stained organoid sections and (**c**,**f**) cytokeratin 5/6 and thrombomodulin double stained organoid sections. Scale bars for panels a–f are 20 μm. For both, panel (i) shows the double stain with DAPI-stained nuclei and panel (ii) shows the double stain with the DAPI channel removed, thereby highlighting colocalization of biomarkers (orange). (**g**,**h**) Representative images (in this case subject 2 organoids) at increased resolution showing the yellow-red overlapping expression of the green channel (ck5/6) and the red channel (calretinin or thrombomodulin). Scale bars for panels g,h are 10 μm.

Tumor organoids from both subjects were assessed by histological methods to verify viability and presence of biomarkers associated with mesothelioma. Imaging of tumor constructs with LIVE/DEAD stain after 7 days (Fig. 2a and d) indicated that viable (green-stained) cells far outweighed dead (red-stained) cells within the construct. Double-stained sections were obtained to highlight colocalization of CK5/6, a high molecular weight keratin, with both calretinin, a calcium binding protein closely linked to mesothelioma (Fig. 2b and e), and with thrombomodulin (also called CD141), a well-established marker for mesothelioma^27–30^ (Fig. 2c and f). Observation of strong overlap with non-selective nuclear stain DAPI confirmed that these constructs retained the mesothelioma phenotype.

### *In vitro* chemotherapeutic drug screens correlate with subject drug response

**Figure 3:**
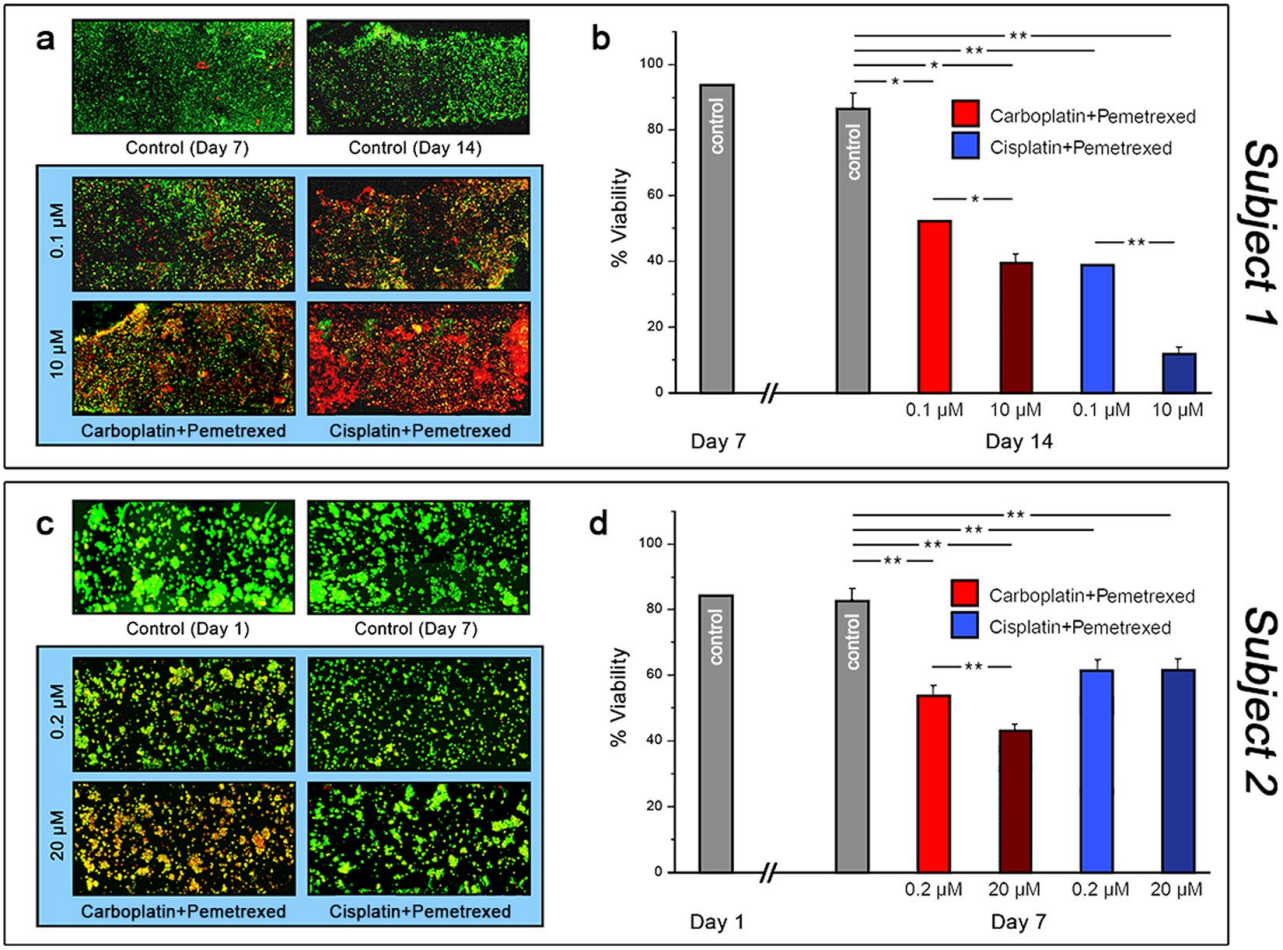
*In vitro* chemotherapy assessment in organoids derived from mesothelioma patients. Maximum projection confocal images of LIVE/DEAD-stained tumor constructs for subject 1 (**a**) and subject 2 (**c**) under conditions indicated. Green: Calcein AM-stained viable cells; Red: Ethidium homodimer-stained dead cells. Analyses of drug-treated constructs collected on day 14 (**a**) and day 7 (**c**). Viability (% live cell count) measurements derived from images for all conditions for subject 1 (**b**) and subject 2 (**d**). Significance: *p < 0.05, **p < 0.01.

For both subjects, a series of constructs were initially kept under DMEM circulation for viability assessment. At the first time point (day 7 for subject 1 and day 1 for subject 2, due to relative growth kinetics of each population), viability analysis was performed on one construct from each subject (Fig. 3a and c, top left). We observed that the vast majority of cells were alive in each sample (93.3% and 84.5%, respectively), confirming that our device architecture could support patient-derived cells. On the same day, circulation to the remaining tumor constructs was either changed to one of two different doses of chemotherapeutic mixtures carboplatin/pemetrexed or cisplatin/pemetrexed, or maintained with no drug as a control. Following an extended exposure, LIVE/DEAD analysis was performed on all organoids. Under control conditions, tumor constructs from both subjects were observed to maintain high viability: subject 1 organoids showed a statistically insignificant decrease to 87% live cells after a total of 14 days in the system (Fig. 3a, top right), while subject 2 organoids showed 83.1% live cells after a total of 7 days in the system (Fig. 3c, top right). These results demonstrated an ability to maintain tumor organoid viability *in vitro*. However, organoids exposed to drug mixtures showed differential reductions in live cell ratio (Fig. 3a and c, bottom). For subject 1 organoids treated with carboplatin/pemetrexed (0.1 μM/0.1 μM or 10 μM/10 μM), we found viabilities reduced to 52.1% for a circulating concentration of 0.1 μM and to 39.8% for 10 μM. While the high dose was two orders of magnitude greater concentration than the low dose, the observed change in viability indicated a potentially moderate effect of the drug. In contrast, organoids treated with cisplatin/pemetrexed (0.1 μM/0.1 μM or 10 μM/10 μM) yielded decreases in viability to 39.0% for 0.1 μM concentration and to 11.8% for 10 μM. These represented significant declines relative both to each other (p < 0.05) and to control measurements (p < 0.05 and p < 0.01, respectively). Consequently, cisplatin/pemetrexed was considerably more effective than carboplatin/pemetrexed in our *in vitro* system. Interestingly, subject 2 organoids displayed a differing drug response, thereby illustrating patient-to-patient tumor heterogeneity. Specifically, subject 2 organoids did not respond significantly to cisplatin/pemetrexed (0.2 μM/0.2 μM or 20 μM/20 μM) in a dose dependent manner, with viabilities reduced to 62.2% for a circulating concentration of 0.1 μM and to 62.5% for 10 μM. Conversely, subject 2 organoids responded to carboplatin/pemetrexed (0.2 μM/0.2 μM or 20 μM/20 μM), which yielded decreases in viability to 54.5% for 0.1 μM concentration and to 43.7% for 10 μM, similar to patient 1.

These results have two central outcomes. First, they demonstrate that cells derived from patient tumor biopsies can be sustained in a bioengineered organoid system long-term for extensive study and, importantly, that they maintain their mesothelioma phenotype. To our knowledge, this is the first report of this capability. Second, they show that *in vitro* drug screening of such organoids is feasible, yielding drug-dependent reductions in cellular viability. This represents a potential asset to therapy design, as drug efficacy could in principle be established in a patient-specific manner prior to administration of treatment. For example, in these measurements, our results with subject 1 organoids would suggest cisplatin/pemetrexed as a superior treatment option to carboplatin/pemetrexed. The nature of personalized medicine prevents a systematic comparison of the effectiveness of the two agents in the patient. Crucially, subject 1 was treated with cisplatin/pemetrexed with excellent response. Conversely, subject 2 had no significant response to cisplatin therapy. This is also observed in our organoid platform experiments and suggests that patient-specific drug effectiveness may be recapitulated accurately in our system. Additionally, our approach supports inter-patient heterogeneity of drug response. For example, in subject 2 organoid drug screens, we observed a more effective drug response to carboplatin/pemetrexed therapy, rather than cisplatin/pemetrexed therapy. This result demonstrates that drug responses measured *in vitro* are not a function of the system, which was identical for subjects 1 and 2, but rather dependent on individual susceptibilities. Notably, it is often the case clinically that drug responses can vary wildly despite patients having similar tumor types or grades, thus illustrating the need for improved personalized medicine approaches.

### *In vitro* drug screening driven by genetic biomarker identification

**Figure 4:**
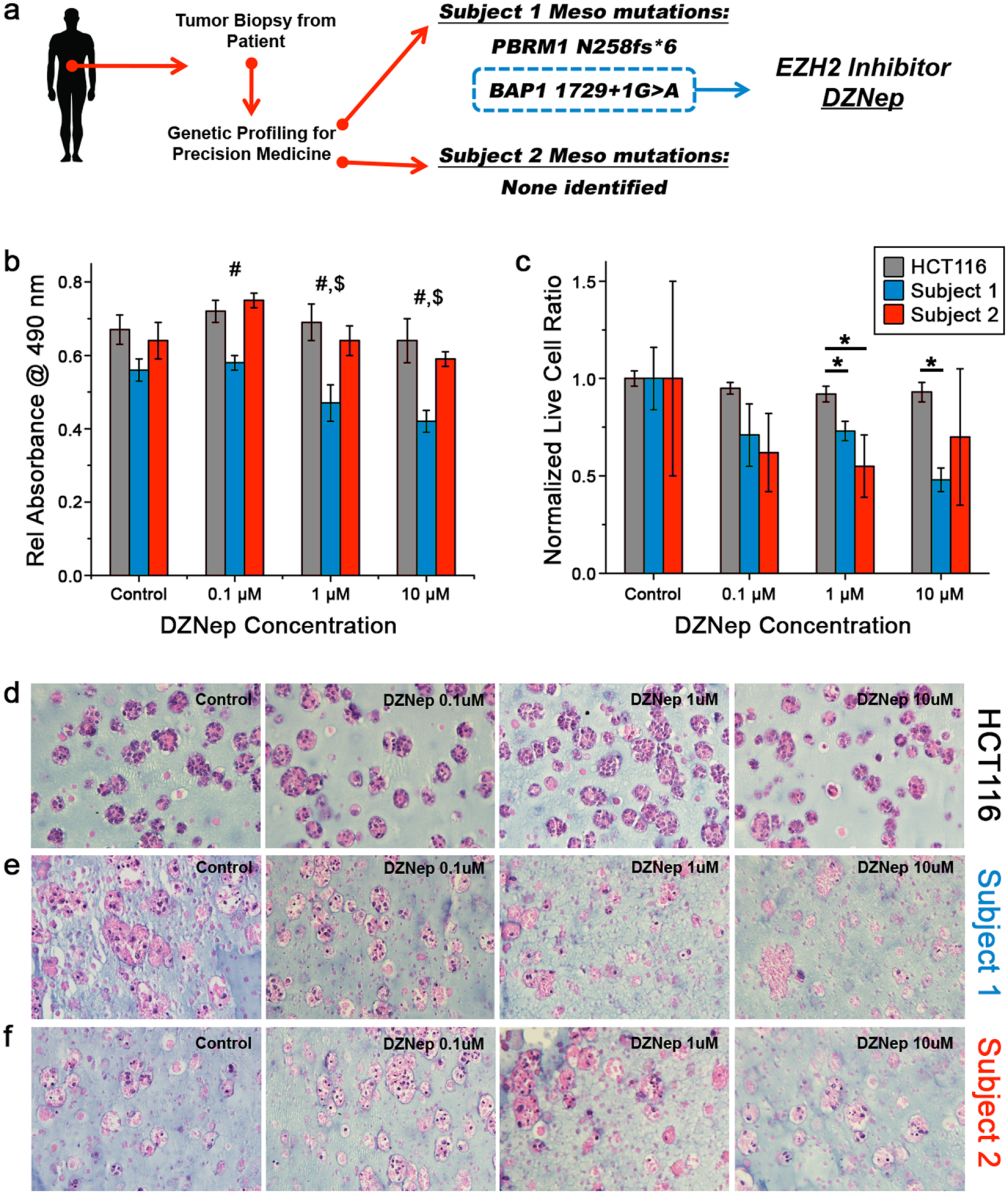
Biomarker-driven therapeutic agent screening in patient-derived tumor constructs. (**a**) Genetic analysis of the tumor biopsy specimen from mesothelioma subject 1 identified two mutations, PBRM1 and BAP1. BAP1 mutations were identified as targetable by the EZH2 inhibitor DZNep. No mutations were identified in the tumor of mesothelioma subject 2. (**b**) MTS mitrochondrial metabolism assay and (**c**) viable/dead cell ratios for HCT116 organoids (white), patient-derived mesothelioma 1 organoids (grey) and patient-derived mesothelioma 2 organoids (dark grey) treated with DZNep concentrations. Control mesothelioma 1 organoids were most proliferative in comparison to those treated with DZNep, which resulted in decreased mitochondrial metabolism (i.e. decreased cell number). Conversely, mesothelioma 2 organoids did not show as significant a response to DZNep, while HCT116 organoids did not respond to DZNep. Significance panel b: ^#^p < 0.05 between subject 1 organoids and both HCT116 and subject 2 organoid response; ^$^p < 0.05 between subject 1 organoid response at 1 μM or 10 μM versus control or 0.1 μM conditions; Significance panel c: *p < 0.05. (**d**–**f**) Hematoxylin and eosin stained organoid sections show loss of normal morphology and appearance of ghost cells with no nuclei as the DZNep concentration increases in the mesothelioma 1 organoids. Mesothelioma 2 and HCT116 organoids do not show the same dose dependent change, suggesting DZNep more effectively induces cell death in the mesothelioma organoids with the BAP1 mutation.

Subject 1 was entered into the Precision Medicine Program at the Comprehensive Cancer Center at the Wake Forest Baptist Medical Center for identification of potential actionable mutations. Results identified two known mutations in the tumor biospecimen: *BAP1* splice site 1729 + 1 G > A and *PBRM1* N258fs*6. *BAP1* (BRCA1 associated protein-1) is a deubiquinating enzyme^31^ while *PBRM1* is a tumor suppressor gene associated with several cancers^32^. At the time of testing, neither mutation was associated with a therapy approved by the FDA for either mesothelioma or any other tumor type. However, the positive identification of the *BAP1* gene mutation provided an opportunity to test an experimental targeted therapy: inhibition of enhancer of zeste homolog 2 (EZH2)^33,34^, a DNA methyltransferase and transcriptional repressor. Importantly, mutations of *BAP1* have been targeted with success in animal models and *in vitro* studies by inhibition of EZH2^33^. This suggests a potential route to PM therapy design (Fig. 4a).

Additional subject 1 tumor constructs were biofabricated and maintained for 3 days prior to drug exposure. Organoids were then subjected to DZNep, a histone methyltransferase EZH2 inhibitor, at concentrations 0.1 μM, 1 μM, and 10 μM in cell culture media for an incubation periods of 96 hours. Drug effects were quantified by MTS assays, IHC staining of Annexin V versus Ki67, and H&E staining. As a control population, identical organoids were also generated using the widely studied colorectal cancer cell line HCT116. Importantly, HCT116 cells have been shown to undergo cell cycle arrest under DZNep exposure, but insignificant cell death^35^, and so they were viewed as an appropriate control for this study.

**Figure 5:**
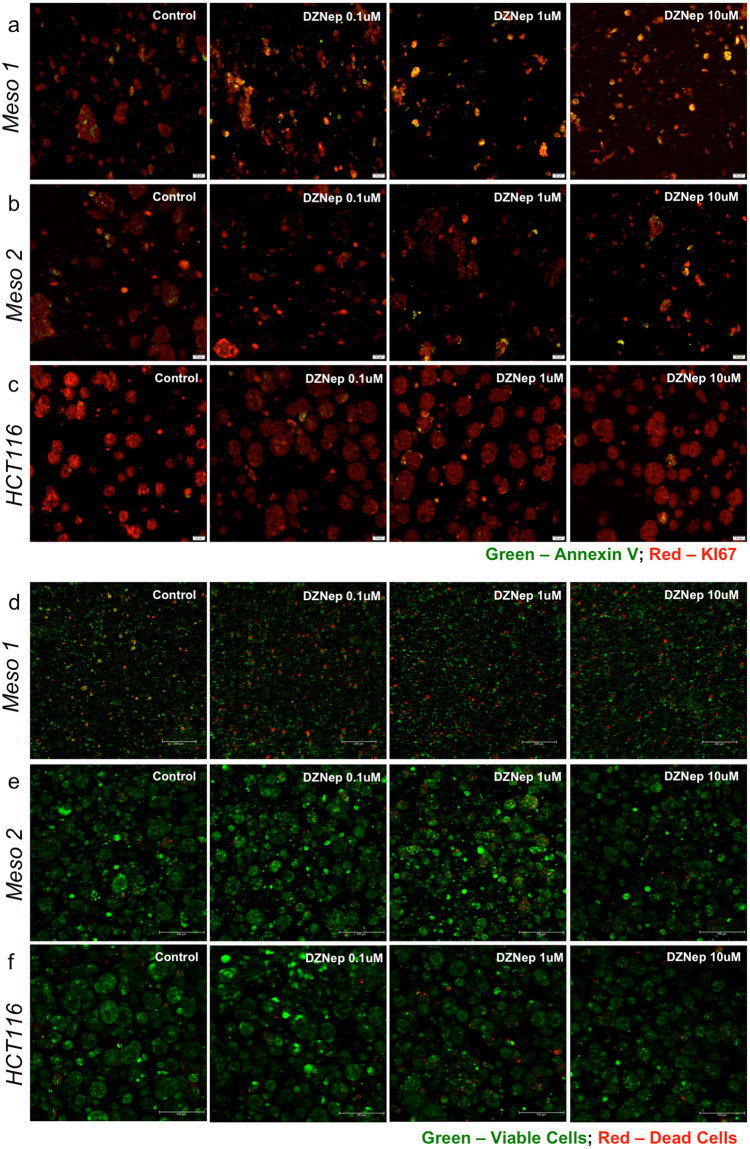
Viability assessment of DZNep-treated mesothelioama 1, mesothelioma 2, and HCT116 organoids. (**a**–**c**) Representative Annexin V and Ki67 biomarker images. Increased observance of yellow/orange signal indicates presence of Annexin V presence overlaid with the red channel. Red stain: KI67; Green stain: Annexin V. (**d**–**f**) LIVE/DEAD assay. Green: Calcein AM-stained viable cells; Red: Ethidium homodimer-stained dead cells. Scale bars are 100 μm.

Our results showed no significant decrease in HCT116 mitochondrial metabolism after DZNep treatment, but a significant decrease in mesothelioma mitochondrial metabolism under all drug concentrations (Fig. 4b). Although viability was similar for both HCT116 and mesothelioma without drug, the DZNep treated mesothelioma showed a concentration dependent decrease in metabolism and in relative live cell percentages (Fig. 4b,c). H&E staining of DZNep-treated subject 1 organoids showed both ghost cells and cells without nuclei, indicating cell death (Fig. 4d), whereas the same effect was not observed in mesothelioma controls or in any HCT116 organoids (Fig. 4f). This response further indicated that DZNep reduced proliferation of the patient-derived sample specifically. Additionally, immunohistochemistry indicated that Annexin V levels increased in mesothelioma with DZNep concentration, representing an increase in apoptotic cells (Fig. 5a). However, minimal change in proliferation was seen across all HCT116 conditions, indicating that DZNep did not significantly affect proliferation (Fig. 5b). Likewise, LIVE/DEAD staining further showed patient 1 organoid susceptibility to DZNep, while minimal cell death was observed in HCT116 organoids (Fig. 5d,f).

In comparison, no actionable mutations were identified by the Precision Medicine Program tumor analysis for subject 2. When subjected to the same DZNep drug screen, subject 2 organoids did not display a decrease in metabolism (Fig. 4b), and displayed a less pronounced decrease in live-to-dead cell ratio compared to subject 1 (Fig. 4c). Stained images of treated subject 2 organoids showed results more akin to the largely DZNep-resistant HCT116 organoids than the subject 1 organoids: nuclei were intact in H&E stains (Fig. 4e), only a small increase in Annexin V expression was observed (Fig. 5c), and there was no significant increase in numbers of dead cells in LIVE/DEAD stained organoids (Fig. 5e)

## Discussion

Precision oncology, whereby tumor DNA is sequenced to identify actionable gene mutations, is poised to become a standard clinical practice for therapeutic decision making of cancer treatment^36–38^. Currently, however, only 11% of precision medicine tested patients will follow a precision medicine guided treatment^39^ because any expected correlation between a genetic alteration and a potential drug cannot be verified prior to initiation of treatment. Moreover, the biological behavior of cancer varies tremendously based on histologic type, grade and volume of disease. Therefore, novel technologies capable of extending the diagnostic utility of tissue specimens are critically needed for robust assessment of genetic alterations and validation of these alterations as actionable targets.

As a research tool, patient derived xenografts (PDX) have been used to study patient tumor progression and drug treatment response. However, a challenge of these models is that they must be hosted by immunodeficient mice and can thus become infiltrated with cells from the mouse, perturbing their natural states. Patient cells also adapt to their new environment and exhibit genetic drift, adding variability. However, the most fundamental limitation to widespread adoption of PDX technology is that only the most aggressive tumor biospecimens prosper in immunodeficient mice. This poor take rate limits the application of PDX technology to predictive diagnostics for the majority of cancer patients. A major open question for *in vitro* platforms like the one described here has been whether the low take rate observed in PDX (and equally in conventional 2D cell culture) could be overcome. Crucially, this can be achieved easily in our platform, where we can obtain high cell viability in organoids over at least 14 days (c.f. Fig. 3a). Moreover, in accompanying studies using identical techniques to produce organoids from additional mesothelioma biospecimens and other GI tumor biospecimens (data not shown), we have observed a take rate above 80%. Beyond high viability, we have also confirmed maintenance of the key mesothelioma biomarkers CK5/6, calretinin, and thrombomodulin in patient-derived organoids (c.f. Fig. 2), showing that our approach supports a degree of accurate tumor phenotype. These results are the first of their kind and suggest that the use of 3D organoid technology incorporating ECM-mimicking microenvironments may be deployable as accurate tumor models for more patients than PDX. Further studies of platform robustness, reproducibility, and take rate efficiency are currently under way.

Using our patient-specific tumor-on-a-chip technology, we demonstrated the feasibility of two central concepts: 1) tumor organoid drug response; and 2) biomarker-driven testing of an experimental drug. First, with mesothelioma biospecimens from 2 different subjects with mesothelioma, we employed the organoid platform to screen two common chemotherapy regimens used clinically for mesothelioma. Specifically, we tested response to the combination of cisplatin and pemetrexed versus carboplatin and pemetrexed (Fig. 3). Exposure to each drug for 7 days through infusion into circulating media via the microfluidic system yielded a distinctly higher efficacy for cisplatin/pemetrexed as compared to carboplatin/pemetrexed at the same concentrations in organoids derived from subject 1. On its own, this result validates the utility of the platform for performing drug screening studies in general. But importantly, the result is also consistent with clinical outcomes; the patient donor responded dramatically to cisplatin-based chemotherapy, presenting almost complete resolution of voluminous ascites on CT imaging performed at the conclusion of cisplatin-based chemotherapy. This kind of significant correlation between patient and *in vitro* model is crucial to validate a methodology for clinical adoption. Additionally, we demonstrated an aspect of patient-to-patient tumor heterogeneity. Specifically, while subject 1 organoids responded best to cisplatin/pemetrexed, subject 2 organoids responded more effectively to carboplatin/pemetrexed. This is an important result, as differential responses to the same therapies are often observed in the clinic. Moreover, subject 2 organoids did not respond significantly to cisplatin-based therapy, matching what was observed *in vivo*. While we consider only two subjects in this foundational study, these striking similarities to subject responses speak to the potential of our platform technology to provide relevant information for clinical practice. Continuing work will further validate this concept as additional patient samples are employed.

Second, genetic testing of the tumor cells from subject 1 identified two mutations specific to the studied tumor: *BAP1* splice site 1729 + 1 G > A and *PBRM1* N258fs*6. As described above, *BAP1* is a deubiquinating enzyme^31^ while *PBRM1* is a tumor suppressor gene associated with several cancers^32^. At the time of these studies, neither mutation was associated with a FDA-approved therapy, either in mesothelioma or in any other tumor type, nor were any pertinent clinical trials under way. However, mutations of *BAP1* have been targeted in animal models and *in vitro* studies by EZH2 inhibition^33^, and so we queried organoid drug response to the EZH2 inhibitor DZNep as a demonstration vehicle for whether the identified mutation could be used as an actionable target (Figs 4 and 5). Indeed, upon treatment with this compound, we observed a decrease in mitochondrial metabolism, which is proportional to cell number. Additionally, we observed an increasing trend in Annexin V expression with drug exposure, indicating apoptosis, as well as a decreasing trend in Ki67 expression, which is present in proliferating cells. Moreover, H&E staining showed that cell morphology and cell nuclei deteriorate in a dose dependent manner. Conversely, organoids created in an identical fashion with the HCT116 colorectal cancer cell line showed no such response regardless of DZNep concentration. While only a single example of biomarker-driven drug screening, this result illustrates the potential of the overall approach to identify novel drugs and experimental agents that benefit the patient prior to administration of treatment. However, we then built on these results from the subject 1 organoids, with organoids from subject 2. Notably, no actionable mutations were identified in the tumor cells of subject 2. In this case, a drug screen with DZNep showed no beneficial response using this drug that is specific to a particular mutation.

A key feature of our platform is integration with a microfluidic delivery system. In these foundational studies, we have employed only moderate parallelization, but the overall approach could be easily expanded to study a wider range of drugs, drug combinations, and exposure doses. The form factor of such devices also enables direct cellular imaging, which in addition to viability assessment could be exploited for tracking the kinetics of tumor progression, migration, and intravasation into circulation. Additionally, the platform opens a route to evolve the tumor-on-a-chip device to a multi-organoid metastasis-on-a-chip device, thereby allowing analysis of tumor progression all the way to distant metastatic sites. We have demonstrated such a system using cell lines^23^, and are currently working to incorporate patient-specific biospecimens.

In this report, we deliberately limit our studies to a small number of drugs to establish two central capacities of our organoid platform technology: namely, performing chemotherapeutic drug screens that recapitulate outcomes in corresponding subjects, and showing statistically significant targeted drug efficacy based on genomic profiling data of the original tumor. These foundational measurements necessitated a relatively small number of organoids. However, we note that a typical clinical tissue sample – especially from a high grade, high volume tumor – could conceivably support a considerably larger number of organoid structures, enabling diverse drugs and drug combinations to be tested in parallel, and potentially informing clinical treatment.

While personalized tumor organoid technology is in its infancy, it holds incredible clinical potential. Once validated through correlation of additional patient-derived organoid drug responses with clinical outcomes, wide adoption of such systems may be able to significantly improve outcomes of cancer patients and reduce unnecessary health care costs through quick matching with the best available effective drugs at the single patient level.

## Materials and Methods

### Experimental design

No animals were employed in the studies performed. Human patient tumor biospecimens were acquired for the study and handled according to a Wake Forest Baptist Medical Center Institutional Review Board-approved protocol (# IRB00040474). All subsequent methods were performed in accordance with the relevant guidelines and regulations. In general, experimental groups consisted of n = 3 or larger to provide robust statistical analysis. Often these experimental groups consisted of two actual experiments for validation of the data. In these controlled *in vitro* studies, no outliers (defined as data points two or more standard deviations from the statistical mean) were observed. Time points for ending studies and ending data collection were defined prior to all experiments, and were not altered during the course of any experiments.

The overall objective of this set of studies was to demonstrate the feasibility of biofabricated patient-derived tumor organoid technology for supporting current efforts in personalied precision oncology. For this technology to be considered for deployment in clinical practice several important criteria need to be met by tumor organoid technology. First, tumor organoids created from patient tumor biospecimen-derived cells must be viable and express biomarkers of the original tumor. Second, the response of organoids to drugs should correlate appropriately with the response of the patient from which the organoids were derived if treated with the same drugs. Third, inter-patient tumor heterogeneity should be supported by this technology. In other words, tumors from different patients may respond to panels of drugs differently, and this heterogeneous response needs to be recapitulated in the organoid studies. Lastly, as precision oncology currently seeks to personalize therapies based on tumor genetic mutations, when such mutations are identified in the tumor, organoids from that tumor should respond when subjected to drugs that exploit those mutations. These criteria are critical for acceptance of personalized tumor organoid technology to succeed, and thus, guided the experimental design of these studies.

### Tumor biospecimen procurement and cell processing

As described, human patient tumor biospecimens were acquired for the study and handled according to a Wake Forest Baptist Medical Center Institutional Review Board-approved protocol (#IRB00040474). Under this protocol, subjects give informed consent for resected tumor tissue removed during CRS HIPEC to be employed in the study. The tumor biospecimens were delivered within one hour of removal to the lab for cell processing. Once received, the sample was washed in phosphate buffered saline (PBS) with 2% penicillin-streptomycin for three 5 min cycles and then washed in Dulbecco’s Modified Eagle’s Medium (DMEM) with 2% penicillin-streptomycin for two 5 minute cycles. The tumor was minced and placed into a conical containing DMEM with 10% collagenase/hyaluronidase (10× Collagenase/Hyaluronidase in DMEM, STEMCELL Technologies, Seattle, WA) and 2% penicillin-streptomycin for 18 hours on a shaker plate in 37 °C. The digested tumor was then filtered through a 100 μm cell filter and centrifuged to create a pellet. Plasma and non-cell material was removed and the pellet was re-suspended in 1 mL BD PharmLyse (BD PharmLyse, San Diego, CA) and 9 mL deionized water for 5 minutes. The mixture was diluted to 50 mL with deionized water and centrifuged, lysis buffer with lysed cells was aspirated, and the cell pellet was counted for use.

### Extracellular matrix (ECM) hydrogel preparation and basic organoid formation

The ECM-mimicking HA/gelatin-based hydrogel (HyStem-HP, ESI-BIO, Alameda, CA) was prepared as previously described^15,18,23^. Briefly, a thiolated HA component (Glycosil) and a thiolated gelatin component (Gelin-S) were dissolved in sterile water containing 0.05% w/v of the photoinitiator 2-Hydroxy-4′-(2-hydroxyethoxy)-2-methylpropiophenone (Sigma, St. Louis, MO) to make a 1% w/v solution. The polyethylene glycol diacrylate (PEGDA) crosslinker (Extralink, ESI-BIO) was dissolved in the photoinitiator solution to make a 2% w/v solution. Glycosil, Gelin-S, and Extralink were then mixed in a 2:2:1 ratio by volume, respectively. For tumor construct formation, the resulting solution was mixed thoroughly by vortexing and then used to resuspend cells (20 million cells/mL).

### Adhesive film microfluidic device fabrication

The microfluidic device fabrication approach (Fig. 1b) is based on methods reported elsewhere^40^. Independently-addressable channels leading to and from individual sample chambers were produced in an adhesive film (cat.# 9495MPF, 3 M, St. Paul, MN) using a cutting plotter (CE6000-40, GraphTec, Tokyo, Japan) and the bottom surface of the resulting patterned layer was attached to a clean glass microscope slide. On the top surface was adhered a polystyrene slide (cat.# 260225, Ted Pella, Redding, CA) featuring holes produced by drill press to act as inlets and outlets for fluid delivery to the channels. Following device assembly, PTFE tubing was connected to each port and secured using a UV cure polyester resin (Solarez, Vista, CA).

### Tumor-on-a-chip biofabrication

Tumor constructs were biofabricated (Fig. 1c) in the microfluidic devices using a photopatterning technique similar to our previous work^15^. Here, a photomask featuring 1–3 mm apertures was produced in an aluminum foil/adhesive film bilayer with the same cutting plotter technique used for channel definition and adhered directly to the bottom surface of the microfluidic device. The mixture of hydrogel precursor solutions and tumor cells derived from the mesothelioma biospecimen was prepared at a density of 20 million cells/mL and then introduced to each of the device channels via the inlet ports and organoid constructs were defined by ultraviolet light exposure (365 nm, 18 W cm^−2^) for 1 s through the attached photomask. This exposure initiated a rapid thiol-ene stepwise crosslinking reaction that encapsulated cells in a 3D column defined by the photomask aperture. Finally, unexposed precursor/cell mixture was flushed from the device with clean PBS, leaving discrete 3D patient-derived mesothelioma constructs. Each channel of the device, featuring one cell construct, was connected to a separate reservoir of DMEM. The media was flowed through the device (Fig. 1d) from the reservoir by silastic tubing connected to MP2 Precision micro-peristaltic pump (Elemental Scientific, Inc., Omaha, NE). Flow was maintained at a rate of 10 μL/min.

### LIVE/DEAD cell viability determination

Circulating media was first flushed from the device channels with clean PBS and then a 1 mL mixture of PBS and DMEM (1:1) containing 2 *μ*M calcein-AM and 2 *μ*M ethidium homodimer-1 (LIVE/DEAD Viability/Cytotoxicity Kit for mammalian cells, ThermoFisher, Waltham, MA) was introduced. Constructs were incubated for 1 hour, after which the channels were again flushed with clean PBS. *In situ* imaging was performed using either a Leica LCS TSI macro-confocal microscope or an Olympus FluoView™ FV1000 confocal microscope. For the latter, 5 μm z-stacks were obtained for each construct using filters appropriate for red and green fluorescence, respectively, and then overlaid. Image quantification was performed using Imaris MeasurementPro sotware (Bitplane, Concord, MA), through which images in each color channel were analyzed for cell size, shape, and fluorescence intensity and cell locations were marked through segmentation (Supplementary Figs 1 and 2). For consistency, a 450 μm × 900 μm area of each image was considered. Cell viability was calculated through quantification of the total number of identified cells in the green channel (LIVE) compared to the total number in the red channel (DEAD).

### Drug treatments

The following drugs were employed in tumor organoid drug studies: cisplatin (Sigma), carboplatin (Sigma), pemetrexed (Sigma), and drug 3-deazaneplanocin A (DZNep, SelleckChem, Houston, TX). Cisplatin/pemetrexed and carboplatin/pemetrexed cocktails were reconstituted in DMEM cell culture media with matched platinum agent and pemetrexed concentrations at 0.1 μM and 10 μM or 0.2 μM and 20 μM for administration to organoids. DZNep was reconstituted in DMEM cell culture media at 0.1 μM, 1 μM, and 10 μM for administration to organoids.

### Histological and immunohistochemistry (IHC) staining

5 *μ*m thick organoid sections were created from paraffin-embedded constructs, and then deparaffinized for staining. IHC was used to visualize biomarkers cytokeratin 5/6 (CK5/6), calretinin, and thrombomodulin. Blocking was performed by incubation under Dako Protein Block for 15 minutes. Primary antibodies CK5/6 (Abcam, ab17133, raised in mouse) and calretinin (Abcam, ab702, raised in rabbit) or CK5/6 and thrombomodulin (Abcam, ab109189, raised in rabbit) were applied to the sections on the slides at a 1:200 dilution in Dako Antibody Diluent and incubated at room temperature for 1 hour. Next, secondary Alexa Fluor 488 or Alexa Fluor 594 antibodies with appropriate species reactivity were applied to all samples at 1:200 in Dako Antibody Diluent and left at room temperature for 1 hour (anti-mouse Alexa Fluor 488 and anti-rabbit Alexa Fluor 594, Life Technologies, Carlsbad, CA, A-11070). Sections were then incubated with Dapi for 5 minutes prior to coverslipping. For Annexin V and Ki67 staining (Abcam, Cambridge, MA, ab14196 and ab16667, respectively) in subsequent biomarker-driven experiments, an identical protocol was employed. Fluorescence images were taken using a Leica DM400B Compound Microscope and overlaid for analysis.

### Cell proliferation assays

MTS assays (CellTiter 96 One Solution Reagent Promega, Madison, WI) determined relative cell number by quantifying mitochondrial metabolism. Absorbance values were determined on a Molecular Devices SpectrumMax M5 (Molecular Devices) tunable plate reader system at a wavelength of 490 nm.

### Statistical Analysis

Data were expressed for each experimental group as mean ± SD and statistical significance determined using statistical analysis methods (Origin, OriginLab Corp., USA, or GraphPad Prism, Graphpad Software Inc., USA). An n = 3 or higher was employed for all studies. Histological, immunohistochemical, or fluorescent images presented in figures are representative of their respective experimental groups. One-way ANOVA were employed for multiple comparisons. Student’s t-tests were performed to compare the means of a normally distributed interval dependent variable for two independent groups. Confidence intervals of 95% or better were considered significant.

## Electronic supplementary material

Supplementary Information
