# Supplementary material for: In vitro patient-derived 3D mesothelioma tumor organoids facilitate patient-centric therapeutic screening
Source: Sci Rep. 2018 Feb 13;8:2886. doi: 10.1038/s41598-018-21200-8 (PMC5811529; doi:10.1038/s41598-018-21200-8)
Supplement: Supplementary file 1 — Supplementary Information [file 41598_2018_21200_MOESM1_ESM.pdf]

Supplementary Materials:

In vitro patient-derived 3D mesothelioma tumor organoids facilitate patient-centric therapeutic screening

Andrea R. Mazzocchi[1,2†], Shiny A. P. Rajan[1,2†], Konstantinos I. Votanopoulos[3,4,*], Adam R. Hall[1,2,4,*], and Aleksander Skardal[1,2,4,5,*]

[1]Wake Forest Institute for Regenerative Medicine, Wake Forest School of Medicine, Medical Center, Winston-Salem, NC, 27101, USA.
[2]Virginia Tech-Wake Forest School of Biomedical Engineering and Sciences, Wake Forest School of Medicine, Medical Center Boulevard, Winston-Salem, NC, 27157, USA.
[3]Department of Surgery-Surgical Oncology, Wake Forest Baptist Medical Center, Medical Center Boulevard, Winston-Salem, NC, 27157, USA.
[4]Comprehensive Cancer Center at Wake Forest Baptist Medical, Medical Center Boulevard, Winston-Salem, NC, 27157, USA.
[5]Department of Cancer Biology, Wake Forest School of Medicine, Medical Center Boulevard, Winston-Salem, NC, 27157, USA.

[†]These authors contributed equally to this work
*Correspondence authors, Wake Forest School of Medicine, Medical Center Boulevard, Winston-Salem, NC 27157
E-mail addresses: askardal@wakehealth.edu (A. Skardal); arhall@wakehealth.edu (A. R. Hall); kvotanop@wakehealth.edu (K. I. Votanopoulos)

Supplementary Figures

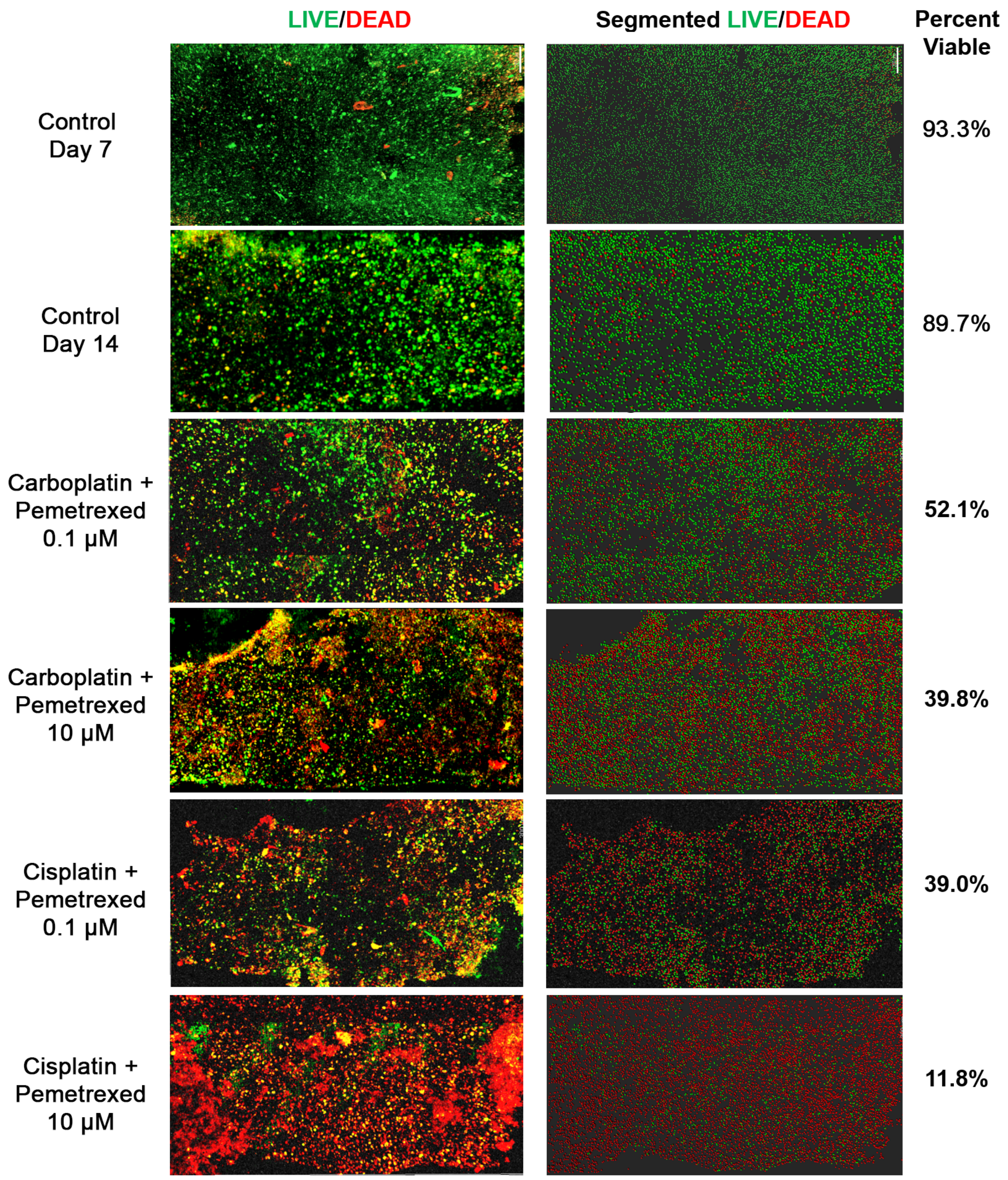

Supplementary Figure 1. Subject 1 organoid LIVE/DEAD segmentation. Same data as in Fig. 3a of the main text, but with segmented images resulting from Imaris analysis software, showing live (green) and dead (red) cell positions.

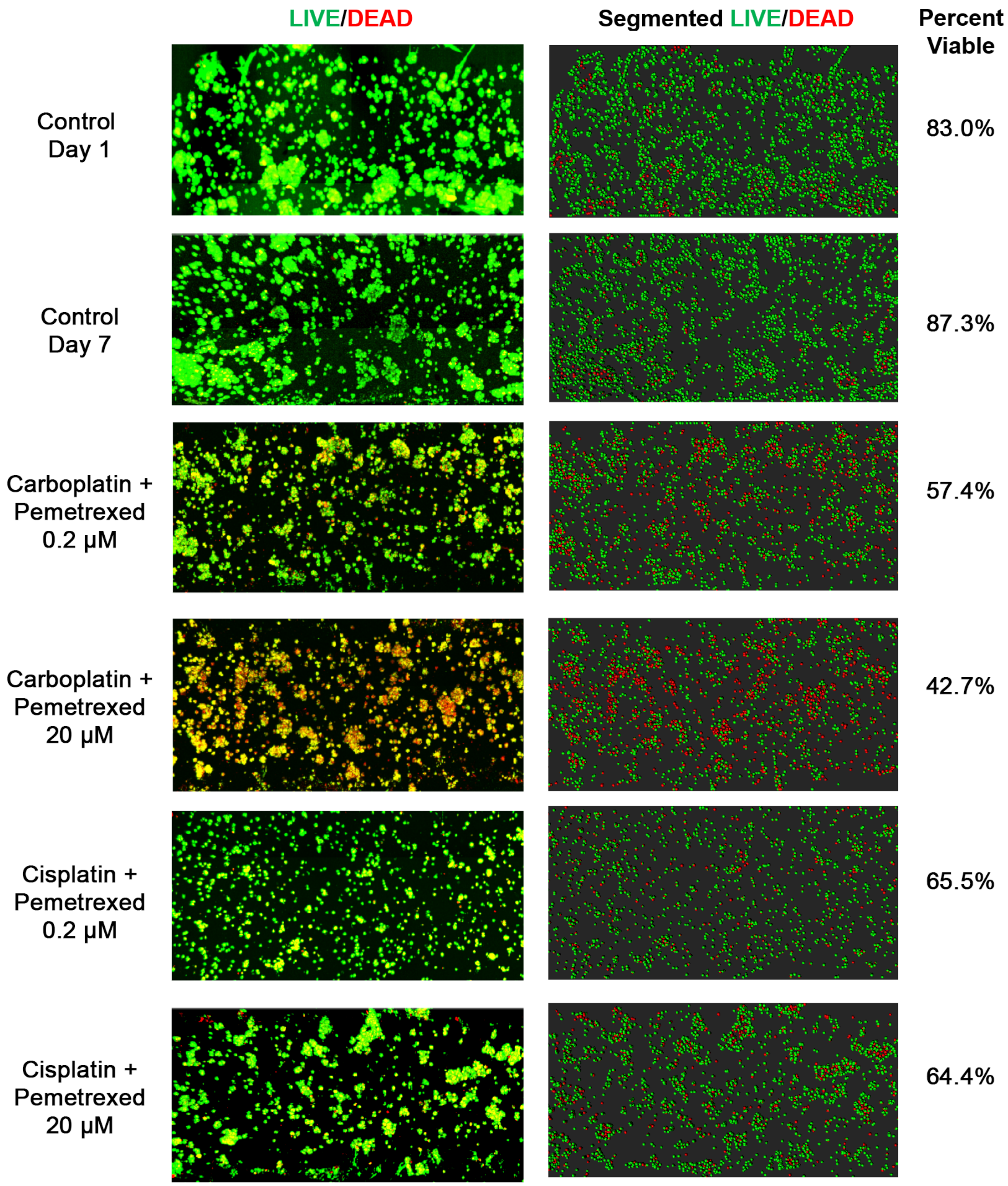

Supplementary Figure 2. Subject 2 organoid LIVE/DEAD segmentation. Same data as in Fig. 3c of the main text, but with segmented images resulting from Imaris analysis software, showing live (green) and dead (red) cell positions.
